# Extracellular vesicles in the Chronic Myeloid Leukemia scenario: an update about the shuttling of disease markers and therapeutic molecules

**DOI:** 10.3389/fonc.2023.1239042

**Published:** 2024-01-08

**Authors:** Simona Bernardi, Olga Mulas, Silvia Mutti, Alessandro Costa, Domenico Russo, Giorgio La Nasa

**Affiliations:** ^1^ Department of Clinical and Experimental Sciences, University of Brescia, Unit of Bone Marrow Transplantation, Azienda Socio Sanitaria Territoriale (ASST) Spedali Civili of Brescia, Brescia, Italy; ^2^ Lab CREA (Centro di Ricerca Emato-oncologica Associazione italiana contro le leucemie, linfomi e mieloma-AIL), ASST Spedali Civili of Brescia, Brescia, Italy; ^3^ Department of Medical Sciences and Public Health, University of Cagliari, Hematology Unit, Businco Hospital, Cagliari, Italy

**Keywords:** Chronic Myeloid Leukemia, extracellular vesicles, exosomes, vesicular markers, therapeutic shuttle, TKIs

## Abstract

Extracellular vesicles (EVs) are various sets of cell-derived membranous structures containing lipids, nucleic acids, and proteins secreted by both eukaryotic and prokaryotic cells. It is now well recognized that EVs are key intercellular communication mediators, allowing the functional transfer of bioactive chemicals from one cell to another in both healthy and pathological pathways. It is evident that the condition of the producer cells heavily influences the composition of EVs. Hence, phenotypic changes in the parent cells are mirrored in the design of the secreted EVs. As a result, EVs have been investigated for a wide range of medicinal and diagnostic uses in different hematological diseases. EVs have only recently been studied in the context of Chronic Myeloid Leukemia (CML), a blood malignancy defined by the chromosomal rearrangement t(9;22) and the fusion gene BCR-ABL1. The findings range from the impact on pathogenesis to the possible use of EVs as medicinal chemical carriers. This review aims to provide for the first time an update on our understanding of EVs as carriers of CML biomarkers for minimal residual disease monitoring, therapy response, and its management, as well as the limited reports on the use of EVs as therapeutic shuttles for innovative treatment approaches.

## 1 Pathogenesis of Chronic Myeloid Leukemia

Chronic Myeloid Leukemia (CML) is a blood cancer characterized by the uncontrolled growth of myeloid cells at different stages of maturation that may be detected both in the bone marrow (BM) and in the peripheral blood (PB) (1). Classically subdivided into three clinical forms, the chronic (CP), accelerated (AP), and blastic phases (BP), the CML represented the pathfinder for many discoveries in the medical domain (2). The translocation t (9;22), also known as Philadelphia (Ph)-chromosome, was identified as the hallmark of CML (3), and the subsequent identified BCR-ABL1 fusion gene is the central player in the pathogenesis of CML. BCR-ABL1 encodes a 210 KD chimeric protein with constitutively active tyrosine kinase activity that promotes several downstream signaling pathways in neoplastic cells (4). In particular, the expression of this oncoprotein leads to altered adhesion to stromal cells and the extracellular matrix, promoting survival and inhibiting apoptosis (3). In addition, cellular transformation and the acquisition of self-renewal capacity are facilitated. Tyrosine kinase inhibitors (TKIs) are the mainstay of current CML treatment. Thanks to their administration, high remission rates have been recorded, and improvements in patient survival rates have been observed. Current guidelines endorse using imatinib, dasatinib, nilotinib, and bosutinib as frontline treatment options in CML patients (5). On the other hand, third-generation TKI ponatinib and newer asciminib are intended for patients previously treated with two or more TKIs or those harboring the T315I mutation (6). Nevertheless, CML continues to be a significant challenge in clinical practice due to the difficulty in predicting its progression and prognosis and the inter-patients variability in CP’s duration and treatment response. Indeed, the presence of the BCR-ABL1 oncoprotein is known to provide for the acquisition of additional genetic abnormalities, likely by increased genomic instability (7). The consequence of this clonal evolution is associated with an increased incidence of relapse, poor prognosis, resistance to TKIs treatment, and, unfortunately, the advancement into blastic crisis (8). The more frequent additional genetic abnormalities detected are duplication of the Ph chromosome, trisomy 8, isochromosome 17, loss of chromosome Y or monosomy 7. In addition, like other hematological malignancies, loss of Tp53 is associated with increased resistance to apoptosis (9, 10). Other important elements in the CML scenario are the leukemic stem cells (LSC) resident in the bone marrow niche. LSC are characterized by the presence of BCR-ABL1 rearrangement and a quiescence that leads to the absence of BCR::ABL1 transcript. The non-transcription of the fusion gene makes LSC undetectable by conventional approaches, such as the quantification of BCR::ABL1 transcript by real-time PCR that is the basis of the minimal residual disease (MRD) monitoring (11, 12). Recently, extracellular vesicles (EVs) and exosomes have generated considerable interest in cancer research (13–15). Increasing evidence suggests that these vesicles are important in regulating immune stimulation or suppression that can drive inflammatory, autoimmune, and infectious disease pathology (16). Given their involvement in disease progression and treatment resistance, EVs have been proven to play an active role in the tumor microenvironment (TME) in the past few years (17, 18). Several findings indicate that they also play a key role in the hematological field and appear to be actively released by LSC. Less is known about their function in CML, especially regarding their potential clinical significance. The purpose of this review is to provide for the first time an overview of the data presented about EVs in CML with a special focus on their role as a shuttle of disease markers and, wherever possible, as new therapeutic approaches.

## 2 The extracellular vesicles

Extracellular vesicles (EVs) are a heterogeneous group of cell-derived membranous structures secreted by both eukaryotic and prokaryotic cells containing lipids, nucleic acids, and proteins. Chargaff and West firstly observed EVs as procoagulant platelet-derived particles in normal plasma in the mid-40s (19). Their presence in various body fluids was then discovered through several studies during the 1970s-80s (20–22). Concomitantly, other researchers observed their origin from tumor masses. The term exosome was born in the same period, referring to vesicles released by multi-vesicular bodies that fuse with the cell membrane. Finally, in the early 2000s, thanks to the evidence that EVs contain nucleic acids, including RNAs such as microRNA (miRNA), EVs acquired substantially renewed interest as players in the cell-to-cell communication (23, 24). Advancing on these pioneering studies, EVs have been resulted as released by most cell types and isolated from different biological fluids (25, 26).

In 2012 The International Society for Extracellular Vesicles (ISEV) was founded. ISEV, including scientists involved in the study of extracellularly secreted vesicles, is considered the reference for the EVs classification and EVs analysis promotion. Based on the current knowledge, EVs can be roughly classified into two main subtypes regarding their physical characteristics and biogenesis pathway: small-EVs (sEVs) and large-EV. The formers have a diameter that ranges between 30–150 nm and include the so-called exosomes. These vesicles derive from intraluminal vesicles formed by the inward budding of the endosomal membrane during the maturation of multivesicular endosomes (MVEs). These are intermediates within the endosomal system and released through the fusion of MVEs with the cell surface. On the other hand, large-EVs represent a heterogeneous population of microvesicles with a diameter that can reach up to 1000 nm. Large vesicles are generated by the outward budding and fission of the cellular lipid membrane and the subsequent secretion of vesicles into the extracellular space (27). As previously stated, EVs are currently established as pivotal mediators of intercellular communication, capable of functionally transferring bioactive molecules from the cell of origin to another in both physiological and pathological pathways. Indeed, released EVs may interact with the releasing cells, therefore acting as autocrine mediators, and with other cell types located both close and far from the cell of origin. Indeed, they act as paracrine or endocrine mediators. EVs exchange information between cells by shuttling several types of molecules, such as proteins, lipids, and the above reported nucleic acids, many of which are selectively sorted inside vesicles (28). Among these regulatory molecules, the miRNAs are expressed by all cell types. Identifying miRNA related to hematological diseases opened up new avenues in biomarkers research. MiRNAs are a family of short, noncoding RNAs that participate in the post-transcriptional regulation of gene expression. They modulate the translation of messenger RNAs (mRNAs) through mechanisms based on the binding of complementary sequences to 3’UTR of mRNA. Their expression is a dynamic process that reflects the evolution of the physiological and pathological condition at the cellular level, which could be an innovative tool in the hematological fields. Indeed, it is well known that they play key roles in almost every cellular process (29).

It is clear that EVs composition largely depends on the status of the producer cells and therefore, one can say that phenotypic alterations in the cell of origin are mirrored by the composition of the secreted EVs, both in terms of EVs type and in terms of cargoes. As a consequence, a multitude of therapeutic and diagnostic applications have been explored for EVs (30, 31). Diagnostic applications take advantage of the different information shuttled by the specific EVs, like the presence of a genetic mutation associated with a disease. On the other hand, therapeutic approaches exploit the EVs capacity to carry and release potentially bioactive molecules (32). In the CML context, the main cells with which the leukemic cell communicates via sEVs are hematopoietic and mesenchymal stem cells, myeloid-derived suppressor cells and endothelial cells (ECs) (33). It is well known that tumor-derived EVs have a remarkable impact on the different recipient cells. Indeed, their effect on cellular proliferation and resistance to apoptosis, induction of angiogenesis, evasion from immune response, transfer of mutations, and modulation of the TME sustains their role as central mediators in key cancer processes. Recent studies confirmed the above-cited pro-tumorigenic activity also in the case of CML-derived EVs. In fact, recent data suggest that they could establish an autocrine loop with their producing cells, through a ligand-receptor interaction mediated by the exosome-associate TGF-β1 (34). Taverna et al. underlined that EVs released from CML cells could affect ECs directly by inducing their release of proangiogenic cytokines, such as IL8, thus modulating neovascularization, which plays an important role in the development and progression of CML (35). The same group has also highlighted that exosomal transfer of miR-126 to ECs directly modulates the adhesive and migratory abilities of CML cells themselves. Other groups reported that the communication between CML cells and surrounding BM stromal cells by CML-derived EVs leads to the inhibition of osteogenesis and thus promotes CML progression. Together, they showed that CML-derived EVs reduce the tumor-suppressive miR-320 in donor cells, resulting in enhanced cell growth *in vivo* models (36). Additionally, CML-derived EVs released by *in vitro* models may transfer the BCR::ABL1 mRNA to normal BM cells, inducing BCR–ABL1 ectopic expression. This intercellular transfer of active biomolecules changes cells’ behavior and promotes disease progression. The disease progression is partially favored by changes in the TME (37) and the immune system’s tolerance. It could be driven by leukemia-derived EVs, as supposed by an Iranian Group who reported that EVs derived by an *in vitro* model of CML drive a tumor-favorable functional performance in T cells (38). This latter evidence has been confirmed by Swatler and colleagues, who demonstrated that leukemic sEVs derived from CML cells promoted leukemia engraftment, associated with an abundance of immunosuppressive T regulatory lymphocytes (Tregs). In the used animal model, the recipient cells changed their transcriptional profile and activated a suppressive activity and effector phenotype by regulating specific receptors’ expression (39, 40). For these reasons, the analysis of sEVs’ features, cargoes, and their potential roles in pathogenesis investigations, patients’ management, and therapy delivery increased the interest of scientists in these small “bullets”.

## 3 sEVs as shuttle of CML markers

As reported above, circulating sEVs cargo has been deeply analyzed to detect potential shuttled leukemia markers (41). Despite the recurring availability of leukemic cells in myeloid leukemias in either BM and/or PB, many groups have conducted studies aiming to improve the sensitivity of the analysis and to reduce the number of invasive and painful BM biopsy (42). Considering the role played by the sEVs and the successful results obtained in the solid tumors by oncologists, these sEVs have also been investigated for their ability to shuttle leukemic biomarkers. DNA, miRNA, mRNA, protein, or lipid profiles associated with different hematologic malignancies are expected to be identified in patients’ sEVs (43). In the CML scenario, the recent insight of circulating sEVs as leukemic biomarkers has highlighted their potential for more sensitive liquid biopsy approaches for an accurate MRD monitoring, a TFR optimization, and an optimal evaluation of drug efficacy. In the following sections, we will critically present and discuss the main results of these pivotal aspects of adult CML patient management.

### 3.1 sEVs for CML MRD monitoring

Thanks to the high efficacy of TKIs targeting BCR-ABL1, the efforts of physicians involved in CML moved from “save the patient” to “monitor the patient” as best as possible. The present strategy for MRD monitoring is based on the quantification of BCR::ABL1 transcript on PB cells, normalized for a reference gene (ABL1 is mainly used). The MRD quantification is internationally standardized and routinely performed by quantitative real-time PCR (RT-qPCR). Two main molecular classes are identifiable. Major Molecular Response (MMR) and Deep Molecular Response (DMR). MMR (also defined as MR3.0) consists of the reduction of the BCR::ABL1 transcript level by at least 3 logs and results in BCR::ABL1/ABL1 ratio < 0,1%. DMR, defined as a BCR::ABL1/ABL1 ratio ≤ 0.01%, can be further subdivided into MR4.0, MR4.5, or MR5.0 when the logarithmic reduction is 4, 4.5, or 5 logs. These reductions are identifiable by BCR::ABL1/ABL1 ratio ≤ 0.01%, ≤ 0.0032%, and ≤ 0.001%, respectively. The sample is considered good quality when ABL1 transcript copies are more than 10.000 at MR4.0, 32.000 at MR4.5, and 100.000 at MR5 (44). These minimums are essential for the definition of the DMR classes in case of undetectable BCR::ABL1 transcript.

Due to the pivotal importance of MRD monitoring in CML patients, sEVs seemed to be very interesting biological tools to support a sensitive, reliable, and relevant detection of resident active leukemic cells. Some years ago, CML researchers questioned the potential role of CML-derived vesicles as disease biomarkers and new sources for the detection of the BCR::ABL1 transcript. The first method relied on the isolation of EVs from the plasma of CML patients via ExoQuickTM Exosome Precipitation Solution and the identification of BCR::ABL1 transcripts based on nested PCR. Even though only patients in the blast and accelerated phases pre-sent BCR::ABL1 transcript within vesicular cargo, vesicular RNA sequence analysis indicated 99% similarity with human cellular BCR::ABL1 (45). Further studies have been carried out as a result of the development of more sophisticated and potent technologies for sEVs isolation and transcript detection, such as dPCR (46, 47). Specifically, it was reported the feasibility of detecting BCR::ABL1 vesicular transcripts in CP-treated CML patients with undetectable MRD levels by standard monitoring (48). The hypothesis of using sEVs content analysis to enhance the detection of active leukemia cells still present in patients’ bodies has been highlighted by this crucial result. Bernardi et al. used a commercial kit immuno-capturing sEVs expressing a pan-cancer antigen to examine the viability of a leukemia-derived sEVs enrichment. Leukemia sEVs enrichment and a BCR::ABL1 transcript detection technique based on dPCR gave the proposed approach a head start (49). The researchers showed for the first time that BCR::ABL1 transcripts could be detected in exosomes circulating in CML patients’ PB, even in cases of patients under TKIs treatment and presenting undetectable MRD levels (50). Moreover, these BCR::ABL1-positive exosomes have been reported as useful in determining the molecular remission grade.

Deviating from the classical MRD monitoring strategy based on the cellular BCR::ABL1 transcript quantification, in the last decade the role of miRNAs in various biological developmental processes and the alteration of their expression was found to broadly influence the phenotype of many cancer subtypes. Many studies have identified hundreds of differentially expressed genes at each stage of the disease using the microarray approach on CML cell lines (51, 52). Flamant and colleagues showed an increased expression of miR-150 and miR-146a, and reduced expression of miR-142-3p and miR-199b-5p in CML cells after 2 weeks of TKI treatment, identifying miRNA as easily measurable biomarkers to monitor the response to TKI (53). Indirectly, it may be considered a measure of viable leukemic cells. Similar recently published results demonstrated that a higher miR-150 and miR-146a expression level predict early response rate in imatinib-treated CML patients (54, 55). Other groups described vesicular miR-29b, miR-320a, miR-30a, and miR-30e as overexpressed in a CML cell line. These miRNA showed to play a role of tumor suppressor, reducing cell proliferation and inducing apoptosis by interfering with BCR-ABL1 activated pathways (56, 57). Moreover, the comparison of vesicular miRNAs between CML patients and healthy subjects highlighted a set of these non-coding RNA differentially expressed in CML patients. This evidence suggests they could play a role in the clinical diagnosis, prognostication, and evaluation of treatment response. For example, the expression of miR-506 and mir451a was shown to be noticeably lower in CML patients than in healthy controls. Moreover, the expression is significantly reduced by the leukemic progression in AP and BP (58, 59). On the other hand, miR-21 increased in CML patients with higher expression in the advanced stages of the disease (60). Some of the main relevant differences in vesicular miRNA expression in CML are recapitulated in **Table 1**.

**Table 1:** Differences in vesicular miRNA expression in CML and their potential implication in clinical practice.

miRNA	EVs Source	Expression	Biomarker	Ref
miR-506	Serum	Down-regulation	Diagnostic, Prognostic	(58)
miR-451a	Plasma	Down-regulation	Prognostic	(59)
miR-21	Blood	Up-regulation	Prognostic,treatment response	(60)
miR-146a	Plasma	Up-regulation	Treatment response	(54)
miR-148b	Blood	Down-regulation	Treatment response	(61)
miR-215	Plasma	Down-regulation	Treatment response	(62)
miR-199b	Plasma	Down-regulation	Treatment response	(63)

EVs, Extracellular Vesicles.

A very interesting breakthrough in the field of sEVs was directed by Valadi and colleagues in 2007. They first reported that exosomes, along with their lipid and protein cargo, contain a significant amount of nucleic acids, particularly mRNAs (24), which lately resulted in translatable into proteins by recipient cells (64). This means that EVs shuttle genetic information. Of note, the term “exosomes” used in the presented studies refers to the EVs classification valid at the time of publication. These new findings, along with the multiple studies regarding the role of miRNAs as disease markers, open the way for exploring vesicular ribonucleic acids as novel reliable disease biomarkers in MRD monitoring.

### 3.2 sEVs in TFR optimization

Among CML patients, a number of them may sustain a TKIs therapy discontinuation, after which they may achieve “treatment-free remission” (TFR) (65). Generally, TKIs discontinuation strategy is adopted in patients that present deep and durable (2 or 3 years) molecular response (DMR), as routinely assessed through RT-qPCR. Nevertheless, many clinical trials demonstrated that no more than 50% can maintain TRF (66). In the last years, it has become clear that the intrinsic limitations of RT-qPCR, among which is the reduced precision in the quantification of the low levels of the target (BCR::ABL1 transcript), are to be considered the main culprit in the erroneous selection of patients eligible for a TKIs discontinuation program. As anticipated, the advent of dPCR opened the way for novel MRD monitoring strategies. dPCR was developed to overcome some of the major limitations of conventional amplification technologies, increasing precision, accuracy, and sensitivity. Bernardi and colleagues underlined that dPCR offers an accurate quantification of BCR::ABL1 transcripts in circulating sEVs, even in patients presenting undetectable MRD levels by conventional monitor. Indeed, it has been proven the capacity of this strategy to improve the detectability of cells releasing BCR::ABL1-positive sEVs. Would this approach support the selection of patients eligible for TKIs therapy discontinuation aiming at TFR? (50). Further studies are needed to answer this question, even if the preliminary results reported in other disease settings are very encouraging (67–69). In order to find potential alternative markers for stopping TKIs use, the role of vesicular miRNA has also been studied. In particular, miR-215 expression was downregulated in the research by Kazuma Ohyashiki et al., both at the cellular and sEVs levels, in CML cases with successful imatinib discontinuation (62). The same authors found that the downregulation of miR-148b had similar effects in TFR patients (61). This data indicates that these miRNA may help with immune surveillance in CML patients with safe TKI discontinuation (61, 62).

Regardless, achieving TFR in a CML setting involves the management of several side effects. Musculoskeletal pain is a common symptom following TKIs discontinuation. However, further insight is still needed to determine the potential contributing variables to this clinical condition. The discovery of a potential main character was made possible by analyzing the exosomal miRNA that circulates in CML patients who have stopped using TKIs. TaqMan low-density array was used to profile exosomal miRNAs, and the results showed that exosomal miR140-3p was substantially elevated in CML patients who reported musculoskeletal pain compared to patients who did not (p = 0.0336) and healthy controls (p = 0.0022). MiR140-3p is thought to have a biological role in inflammation, and CML patients who have experienced symptom relief have substantially lower exosomal levels of the protein. These findings suggest that exosomal miRNA analysis could be used to identify treatment side effects or effectiveness when TKIs are being used (70).

### 3.3 sEVs for therapy efficacy

Despite the TKIs’ above-mentioned remarkable effectiveness in treating CML, a tiny percentage of patients develop drug resistance while receiving TKIs therapy. Physicians are still triggered by it. The BCR-ABL1 protein’s acquired point mutations are primarily linked to TKIs resistance, but little is known about how resistance traits can develop in cells lacking these variants. One of the most recent processes, similar to what has been seen in acute myeloid leukemia cells, is the vesicular-mediated transfer of molecules from resistant to sensitive CML cells (71). In particular, high levels of exosomal miR365 have been reported in cases of lower drug sensitivity and lower apoptosis rate. The exposure of sensitive CML cells to exosomes released by resistant and miR365-rich cells induced drug resistance. This process is due to the inhibition of pro-apoptotic proteins in sensitive CML cells (72). In support of these results, the influence of circulating miRNA in CML-derived cells has been demonstrated even when they are not carried by vesicles. Hershkovitz-Rokah et al. demonstrated the capacity of miR-30e to sensitize K562 cells and patient primary cells to imatinib treatment through regulation of cell cycle progression between G1 and S phases (73). MiR-199b targets HES1, a transcription factor involved in the Notch pathway and highly conserved among multicellular organisms. It regulates cell-fate determination during development and maintains adult tissue homeostasis. Expression studies have revealed downregulation of miR-199b in CML patients presenting 9q deletion. A lower level of miR-199b was found in imatinib-resistant patients, suggesting that it could be considered one of the factors for drug resistance (63).

Moreover, an additional study confirmed that exosomes released by imatinib−resistant K562 (K562IR) cells and internalized by imatinib−sensitive cells of the same line (K562IS) could increase the survival of the latter. This phenomenon was observed even in the presence of toxic doses of imatinib (2 μM). K562IR-exosomes characterization led to three specific cell-surface markers, namely, IFITM3, CD146, and CD36, that resulted in upregulation when compared to K562IS-exosomes. The upregulation of these proteins was later verified in the K562IR cells confirming that sEVs mirror the parental cell’s features. Flow cytometric analysis further demonstrated the potential of CD146 as a cell surface marker expressed by K562 cells presenting imatinib resistance. These results suggest that exosomes and the related membrane proteins could be potential diagnostic markers of drug resistance in CML patients treated with TKIs (74). Conversely, miR328 has been reported as significantly associated with sensitivity to first-generation TKI in another *in vitro* CML model. For instance, *in vitro* delivery of alkalized exosomes, containing or not miR328 as cargo, elevated endogenous miR328 levels, inducing sensitivity to imatinib. Moreover, endogenous miR328 suppression produced imatinib resistance in the K562 CML cell line (75). Similarly, miR-185 expression sensitizes Ph+ cells to TKIs-induced apoptosis and affects their proliferation rate, partly through a BCR-ABL1-kinase-dependent mechanism. Overall, restoration of miR-185 expression had an evident effect on the survival of patient-derived TKI-insensitive stem/progenitor cells isolated in patients and cultured *in vitro* in the presence of TKIs (76). In addition, the imatinib sensitivity of K562 cell line was tested in another trial administering exosomes released by human umbilical cord MSCs (hUC-MSCs) during the cell culture. Exosomes released by hUC-MSCs alone seem to unaffected cell viability but promote imatinib-induced cell death. Moreover, they activate caspase-9 and caspase-3 more than imatinib alone (77). Lately, Chen X. et al., elucidated the role of miR-146a-5p/USP6/GLS1 in leukemia and chemoresistance of leukemia cells and confirmed hUC-MSC exosomes capacity to promote imatinib-induced cell apoptosis through miR-145a-5p/USP6. USP6 levels were elevated and related to a poor prognosis in BM aspiration samples from CML patients. Compared to clinical samples that were imatinib-sensitive, USP6 was markedly increased in imatinib-resistant samples. Leukemia cells’ apoptosis was dramatically reduced by USP6 overexpression in response to imatinib. Increased GLS1 ubiquitination caused by overexpression of USP6 reduced GLS protein. A mechanistic investigation revealed that miR-146a-5p and GLS1 were both required for USP6 control of the imatinib resistance of CML cells. Through miR-145a-5p/USP6, the administration of hUC-MSCs exosomes increased imatinib-induced cell death. Therefore, through miR-146a-5p and its target GLS1, hUC-MSC exosomes increased imatinib-induced death of K562-R cells by decreasing GLS1 ubiquitination and increasing GLS protein. The research sheds fresh information on the role of miR-146a-5p/USP6/GLS1 signaling in leukemia chemoresistance (78).

Little is known about the role of second-generation TKIs. Although no data are reported on the role of vesicles in this context, direct expression of miRNA still appears to have an important place in the interaction of other TKIs besides imatinib. This also supports what above reported and commented. Indeed, the combined expression of different miRNAs was recently investigated in CML cells exposed to nilotinib. Particularly, miR-145 and miR-708 expressions were associated as a predictive indicator of nilotinib response at the treatment-naïve state. In addition, higher expressions of miR-150 and decreased levels of miR-185 were found in nilotinib non-responders, compared with nilotinib responders (79). Liu et al. described one of the possible mechanisms through which dasatinib could be able to overcome imatinib resistance. Their reports highlighted that dasatinib promotes cellular apoptosis by downregulation of Akt/mTOR pathway activities. Moreover, dasatinib prevents exosomal release through the downregulation of beclin-1 and Vps34-dependent autophagic activity. These results suggest distinct dasatinib-induced activation of apoptotic response and exosome generation in CML cells resistant to first-generation TKI (80). Hence, the synergy of exosomes and TKIs may be considered an effective approach to improve the response rate during CML treatment and provide an interesting basis for new therapeutic strategies designed for chemoresistant/target therapy-resistance leukemia.

**Figure 1:**
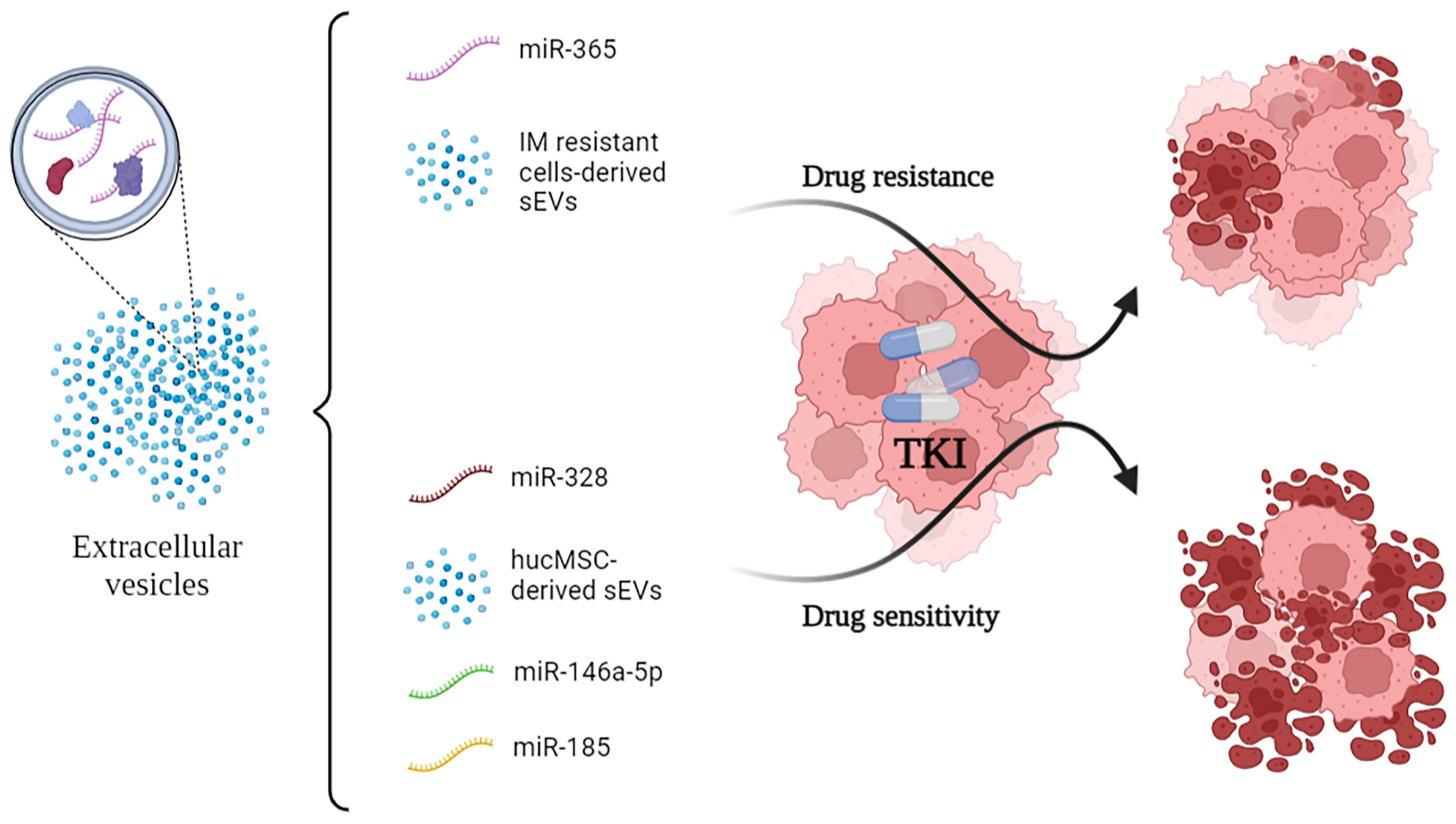
Small-EVs and some of their encapsulated miRNA show opposite effects on the efficacy of TKI administration to CML cells. In particular, vesicular miR-365 drives resistance to TKI in the recipient cells. Conversely, miR-328, miR-146a-5p and miR-185 drive sensitivity to TKI. TKI, Tyrosine Kinase Inhibitors.

All of the cited mechanisms are summarized in **Figure 1**.

## 4 EVs as shuttle of therapeutic molecules

Despite the success of TKIs-based therapy, the exploration of exosome-based therapy, which combines the vesicles with both TKIs and unusual molecules, was surprisingly more prevalent in CML. The target of CML blasts was reported in an outstanding work using modified exosomes loaded with imatinib. HEK293T cell line transfected with plasmids encoding the exosomal protein Lamp2b fused to a portion of interleukin 3 (IL3) was used. The researchers selected this protein because it is known that the IL3 receptor is overexpressed on the surface of CML blasts. It was found that imatinib and BCR-ABL1-silencing RNA could be delivered to CML cells by exosomes produced by the transfected cells. In sensitive and resistant models, as well as *in vitro* and *in vivo* models, this ability resulted in reduced leukemia cell proliferation. For example, in a mouse model, imatinib-laden IL3-exosomes significantly reduced the tumor burden when compared to imatinib-free IL3-exosomes, regular exosomes loaded with imatinib, and imatinib alone (p <0.0005) (81). Similarly, CML exosomes exposed to a TGF-β1 receptor inhibitor or a specific neutralizing TGF-β1 antibody significantly reverse the proliferation of CML cells compared to those exposed to TGF-β1 enriched LAMA84 exosomes (34). These pivotal data strongly support the application of exosomes as specific drug delivery tools even in CML, as observed in TKIs-resistant cells. The authors achieved these impressive results after many evidences obtained by using exosomes for delivery of unconventional molecules *in vitro* models of CML. In particular, the authors reported the impact of curcumin on CML exosome composition (82) and the use of common lemon-juice-derived small vesicles. The latter were able to suppress leukemia proliferation in xenograft models using NOD/SCID mice subcutaneously inoculated with CML cells. On the other hand, alkalized exosomes have been shown to block miR328 lysosomal degradation and thus sensitize CML cells to imatinib (75). In addition, the exosomes specifically reached the leukemic site within the mice model and activated apoptotic cell processes (83). Recently, Cochran et al. showed that Natural Killer-derived exosomes (NKexo) were able to maintain the anti‐leukemia capacity of their donor NK‐cells, NKexo resulted in cytotoxic against malignant hematopoietic cell lines (K562 and Jurkat), thus acting as a potential acellular therapeutic modality (84). Results showed that low doses of NK3.3 EVs inhibited the growth of K562 cells over 72 h, while high doses of NK3.3 EVs were cytotoxic. These findings were verified by Samara’s group’s latest study (85), which additionally provided a more comprehensive analysis of the time‐ and dose‐dependent antileukemic activity of NKexo, on a wider variety of leukemia cell lines and ex-vivo models derived from patients’ samples. Firstly, they showed that NKexo (20 μg) have the ability to increase apoptosis rates by up to 64.37 (± 11.7%) across all biomodels, including K562. In contrast, healthy‐donor PB Mononuclear Cells presented no alteration, suggesting a selective cytotoxic effect targeting leukemia cells. Moreover, NKexo cytolytic activity via the release of cytotoxic effectors was confirmed, and a reduction in cell count, ranging between 65% and 84%, was seen in all leukemia cell lines tested, including K652. Finally, the clonogenic potential of treatment‐naïve CML-derived cells was significantly reduced by 20 μg of NKexo during a 14 days long cell culture. The relative colony‐formation efficiency of CML cells was reduced by an average of 28 ± 14% (p ≤0.005). Similarly, umbilical cord mesenchymal cell-derived exosomes were proven able to promote Imatinib-induced apoptosis in K562-R cells via miR-146a-5p and its target USP6, which suppress GLS1 ubiquitination, causing an increase in GLS protein (78). Zang et al. recently demonstrated that another source of mesenchymal cell-derived exosomes, such as human BM, could inhibit the proliferation of CML cells *in vitro* via miR-15a and arrest the cell cycle in the G0/G1 phase. On the other hand, the same authors found that these mesenchymal exosomes promoted the proliferation and decreased the sensitivity of CML cells to TKIs, resulting in drug resistance in the xenograft tumor model (86). Finally, to assess innovative therapeutic approaches, another very interesting strategy was evaluated to increase treatment outcomes in CML patients. Indeed the authors developed a sophisticated liposome conjugated with Begelomab (anti-CD26) loaded with venetoclax to target CD26+ CML LSCs/progenitor cells selectively. They proved that the CD26+ LSCs/progenitor cells could be eliminated after antigen binding and drug release without any side effects on CD26− cells (87).

## 5 Conclusions

In this review, we have brought together the main knowledge about extracellular vesicles, comprehensively addressing various crucial CML aspects. CML has been the first disease for which a targeted therapy was identified, paving the way for novel treatments in other hematological fields. Over the course of its history, there has been a gradual improvement in monitoring and new goals of treatment, such as TFR, have been attained. Despite few data available, if compared to other cancers, EVs could have multiple applications in CML. Undoubtedly, many efforts have been made to evaluate the role of EVs in treatment response monitoring and encouraging results have been observed in MRD monitoring. It is fascinating to see the possible application of these extracellular bodies into TKIs-resistant disease and their role, such as shuttle for other specific drugs. On the other hand, the lack of standardization, and the large variability in EVs, imply that their use is still limited to often speculative valuations. Further research is needed to understand their role. In summary, this review could be an essential source of knowledge for future studies about EVs as a crucial mediator for new therapeutic strategies in CML.

## Author contributions

Conceptualization, SB and OM; data curation, SB, OM, SM, AC, DR and GL; writing—original draft preparation, SB, OM, SM, and AC; writing—review & editing, SB, OM, SM, AC, DR and GL, supervision, SB, OM, DR and GL. All authors have read and agreed to the published version of the manuscript.

## References

[1] Goldman JM Melo JV. Targeting the BCR-ABL tyrosine kinase in chronic myeloid leukemia. N Engl J Med (2001) 344:1084–6. doi: 10.1056/NEJM200104053441409 11287980

[2] Hochhaus A Dreyling M. ESMO guidelines working group chronic myelogenous leukemia: ESMO clinical recommendations for the diagnosis, treatment and follow-up. Ann Oncol Off J Eur Soc Med Oncol (2008) 19 Suppl 2:ii63–64. doi: 10.1093/annonc/mdn091 18456772

[3] Ren R. Mechanisms of BCR-ABL in the pathogenesis of chronic myelogenous leukaemia. Nat Rev Cancer (2005) 5:172–83. doi: 10.1038/nrc1567 15719031

[4] Perrotti D Jamieson C Goldman J Skorski T. Chronic myeloid leukemia: mechanisms of blastic transformation. J Clin Invest. (2010) 120:2254–64. doi: 10.1172/JCI41246 PMC2898591 20592475

[5] Hochhaus A Baccarani M Silver RT Schiffer C Apperley JF Cervantes F. European leukemiaNet 2020 recommendations for treating chronic myeloid leukemia. Leukemia (2020) 34:966–84. doi: 10.1038/s41375-020-0776-2 PMC7214240 32127639

[6] Cortes JE Kim D-W Pinilla-Ibarz J le Coutre P Paquette R Chuah C. A phase 2 trial of ponatinib in philadelphia chromosome-positive leukemias. N Engl J Med (2013) 369:1783–96. doi: 10.1056/NEJMoa1306494 PMC3886799 24180494

[7] Muller AJ Young JC Pendergast AM Pondel M Landau NR Littman DR. BCR first exon sequences specifically activate the BCR/ABL tyrosine kinase oncogene of philadelphia chromosome-positive human leukemias. Mol Cell Biol (1991) 11:1785–92. doi: 10.1128/mcb.11.4.1785-1792.1991 PMC359845 2005881

[8] Skorski T. Genomic instability: the cause and effect of BCR/ABL tyrosine kinase. Curr Hematol Malig. Rep (2007) 2:69–74. doi: 10.1007/s11899-007-0010-6 20425353

[9] Cortes JE Talpaz M Giles F O’Brien S Rios MB Shan J. Prognostic significance of cytogenetic clonal evolution in patients with chronic myelogenous leukemia on imatinib mesylate therapy. Blood (2003) 101:3794–800. doi: 10.1182/blood-2002-09-2790 12560227

[10] O’Dwyer ME Mauro MJ Blasdel C Farnsworth M Kurilik G Hsieh Y-C. Clonal evolution and lack of cytogenetic response are adverse prognostic factors for hematologic relapse of chronic phase CML patients treated with imatinib mesylate. Blood (2004) 103:451–5. doi: 10.1182/blood-2003-02-0371 14512312

[11] Vetrie D Helgason GV Copland M. The leukaemia stem cell: similarities, differences and clinical prospects in. Nat Rev Cancer (2020) 20:158–73. doi: 10.1038/s41568-019-0230-9 31907378

[12] Arrigoni E Del Re M Galimberti S Restante G Rofi E Crucitta S. Concise review: chronic myeloid leukemia: stem cell niche and response to pharmacologic treatment. Stem Cells Transl Med (2018) 7:305–14. doi: 10.1002/sctm.17-0175 PMC5827745 29418079

[13] Ahmadi M Mahmoodi M Shoaran M Nazari-Khanamiri F Rezaie J. Harnessing normal and engineered mesenchymal stem cells derived exosomes for cancer therapy: opportunity and challenges. Int J Mol Sci (2022) 23:13974. doi: 10.3390/ijms232213974 36430452 PMC9699149

[14] Rezaie J Etemadi T Feghhi M. The distinct roles of exosomes in innate immune responses and therapeutic applications in cancer. Eur J Pharmacol (2022) 933:175292. doi: 10.1016/j.ejphar.2022.175292 36150532

[15] Ahmadi M Jafari R Mahmoodi M Rezaie J. The tumorigenic and therapeutic functions of exosomes in colorectal cancer: opportunity and challenges. Cell Biochem Funct (2021) 39:468–77. doi: 10.1002/cbf.3622 33491214

[16] Robbins PD Morelli AE. Regulation of immune responses by extracellular vesicles. Nat Rev Immunol (2014) 14:195–208. doi: 10.1038/nri3622 24566916 PMC4350779

[17] Szczepanski MJ Szajnik M Welsh A Whiteside TL Boyiadzis M. Blast-derived microvesicles in sera from patients with acute myeloid leukemia suppress natural killer cell function via membrane-associated transforming growth factor-beta1. Haematologica (2011) 96:1302–9. doi: 10.3324/haematol.2010.039743 PMC3166100 21606166

[18] Caivano A Laurenzana I De Luca L La Rocca F Simeon V Trino S. High serum levels of extracellular vesicles expressing Malignancy-related markers are released in patients with various types of hematological neoplastic disorders. Tumour Biol J Int Soc Oncodevelopmental Biol Med (2015) 36:9739–52. doi: 10.1007/s13277-015-3741-3 26156801

[19] Chargaff E West R. The biological significance of the thromboplastic protein of blood. J Biol Chem (1946) 166:189–97. 20273687

[20] Stegmayr B Ronquist G. Promotive effect on human sperm progressive motility by prostasomes. Urol. Res (1982) 10:253–7. doi: 10.1007/BF00255932 6219486

[21] Dalton AJ. Microvesicles and vesicles of multivesicular bodies versus “Virus-like” Particles. J Natl Cancer Inst (1975) 54:1137–48. doi: 10.1093/jnci/54.5.1137 165305

[22] Ronquist G Brody I Gottfries A Stegmayr B. An mg2+ and ca2+-stimulated adenosine triphosphatase in human prostatic fluid. Andrologia (1978) 10:261–72. doi: 10.1111/j.1439-0272.1978.tb03030.x 152589

[23] Ratajczak J Miekus K Kucia M Zhang J Reca R Dvorak P. Embryonic stem cell-derived microvesicles reprogram hematopoietic progenitors: evidence for horizontal transfer of mRNA and protein delivery. Leukemia (2006) 20:847–56. doi: 10.1038/sj.leu.2404132 16453000

[24] Valadi H Ekström K Bossios A Sjöstrand M Lee JJ Lötvall JO. Exosome-Mediated Transfer of mRNAs and microRNAs Is a Novel Mechanism of Genetic Exchange between Cells. Nat Cell Biol (2007) 9:654–9. doi: 10.1038/ncb1596 17486113

[25] Mahbubfam S Rezaie J Nejati V. Crosstalk between exosomes signaling pathway and autophagy flux in senescent human endothelial cells. Tissue Cell (2022) 76:101803. doi: 10.1016/j.tice.2022.101803 35472675

[26] Shaban SA Rezaie J Nejati V. Exosomes derived from senescent endothelial cells contain distinct pro-angiogenic miRNAs and proteins. Cardiovasc Toxicol (2022) 22:592–601. doi: 10.1007/s12012-022-09740-y 35441341

[27] Yáñez-Mó M Siljander PR-M Andreu Z Zavec AB Borràs FE Buzas EI. Biological properties of extracellular vesicles and their physiological functions. J Extracell. Vesicles (2015) 4:27066. doi: 10.3402/jev.v4.27066 25979354 PMC4433489

[28] van der Pol E Böing AN Harrison P Sturk A Nieuwland R. Classification, functions, and clinical relevance of extracellular vesicles. Pharmacol Rev (2012) 64:676–705. doi: 10.1124/pr.112.005983 22722893

[29] Ciafrè SA Galardi S. microRNAs and RNA-binding proteins. RNA Biol (2013) 10:934–42. doi: 10.4161/rna.24641 PMC4111733 23696003

[30] Rezaie J Nejati V Mahmoodi M Ahmadi M. Mesenchymal stem cells derived extracellular vesicles: A promising nanomedicine for drug delivery system. Biochem Pharmacol (2022) 203:115167. doi: 10.1016/j.bcp.2022.115167 35820499

[31] Almohammai A Rahbarghazi R Keyhanmanesh R Rezaie J Ahmadi M. Asthmatic condition induced the activity of exosome secretory pathway in rat pulmonary tissues. J Inflamm Lond Engl (2021) 18:14. doi: 10.1186/s12950-021-00275-7 PMC8015058 33794910

[32] Bernardi S Farina M. Exosomes and extracellular vesicles in myeloid neoplasia: the multiple and complex roles played by these Magic bullets. Biology (2021) 10:105. doi: 10.3390/biology10020105 33540594 PMC7912829

[33] Jurj A Pasca S Teodorescu P Tomuleasa C Berindan-Neagoe I. Basic knowledge on BCR-ABL1-positive extracellular vesicles. biomark Med (2020) 14:451–8. doi: 10.2217/bmm-2019-0510 32270699

[34] Raimondo S Saieva L Corrado C Fontana S Flugy A Rizzo A. Chronic myeloid leukemia-derived exosomes promote tumor growth through an autocrine mechanism. Cell Commun Signal CCS (2015) 13:8. doi: 10.1186/s12964-015-0086-x 25644060 PMC4320527

[35] Taverna S Flugy A Saieva L Kohn EC Santoro A Meraviglia S. Role of exosomes released by chronic myelogenous leukemia cells in angiogenesis. Int J Cancer (2012) 130:2033–43. doi: 10.1002/ijc.26217 PMC3236253 21630268

[36] Gao X Wan Z Wei M Dong Y Zhao Y Chen X. Chronic Myelogenous Leukemia Cells Remodel the Bone Marrow Niche via Exosome-Mediated Transfer of miR-320. Theranostics (2019) 9:5642–56. doi: 10.7150/thno.34813 PMC6735391 31534508

[37] Jiang Y-H Liu J Lin J Li S-Q Xu Y-M Min Q-H. K562 Cell-Derived Exosomes Suppress the Adhesive Function of Bone Marrow Mesenchymal Stem Cells via Delivery of miR-711. Biochem Biophys Res Commun (2020) 521:584–9. doi: 10.1016/j.bbrc.2019.10.096 31677790

[38] Jafarzadeh N Gholampour MA Alivand M-R Kavousi S Arzi L Rad F. CML derived exosomes promote tumor favorable functional performance in T cells. BMC Cancer (2021) 21:1002. doi: 10.1186/s12885-021-08734-3 34493241 PMC8424959

[39] Swatler J Dudka W Bugajski L Brewinska-Olchowik M Kozlowska E Piwocka K. Chronic myeloid leukemia-derived extracellular vesicles increase foxp3 level and suppressive activity of thymic regulatory T cells. Eur J Immunol (2020) 50:606–9. doi: 10.1002/eji.201848051 PMC7187374 31758697

[40] Swatler J Turos-Korgul L Brewinska-Olchowik M De Biasi S Dudka W Le BV. 4-1BBL-containing leukemic extracellular vesicles promote immunosuppressive effector regulatory T cells. Blood Adv (2022) 6:1879–94. doi: 10.1182/bloodadvances.2021006195 PMC8941461 35130345

[41] Gholipour E Sarvarian P Samadi P Talebi M Movassaghpour A Motavalli R. Exosome: from leukemia progression to a novel therapeutic approach in leukemia treatment. BioFactors Oxf. Engl (2020) 46:698–715. doi: 10.1002/biof.1669 32797698

[42] Boyiadzis M Whiteside TL. Plasma-derived exosomes in acute myeloid leukemia for detection of minimal residual disease: are we ready? Expert Rev Mol Diagn. (2016) 16:623–9. doi: 10.1080/14737159.2016.1174578 PMC5400097 27043038

[43] Boyiadzis M Whiteside TL. The emerging roles of tumor-derived exosomes in hematological Malignancies. Leukemia (2017) 31:1259–68. doi: 10.1038/leu.2017.91 28321122

[44] Cumbo C Anelli L Specchia G Albano F. Monitoring of minimal residual disease (MRD) in chronic myeloid leukemia: recent advances. Cancer Manage Res (2020) 12:3175–89. doi: 10.2147/CMAR.S232752 PMC7211966 32440215

[45] Kang K-W Jung J-H Hur W Park J Shin H Choi B. The potential of exosomes derived from chronic myelogenous leukaemia cells as a biomarker. Anticancer Res (2018) 38:3935–42. doi: 10.21873/anticanres.12679 29970515

[46] Soverini S Bernardi S Galimberti S. Molecular testing in CML between old and new methods: are we at a turning point? J Clin Med (2020) 9:3865. doi: 10.3390/jcm9123865 33261150 PMC7760306

[47] Zanaglio C Bernardi S Gandolfi L Farina M Re F Polverelli N. RT-qPCR versus digital PCR: how do they impact differently on clinical management of chronic myeloid leukemia patients? Case Rep Oncol (2020) 13:1263–9. doi: 10.1159/000510440 PMC7670369 33250741

[48] Bernardi S Malagola M Polverelli N Russo D. Exosomes in chronic myeloid leukemia: are we reading a new reliable message? Acta Haematol (2020) 143:509–10. doi: 10.1159/000505088 31922494

[49] Bernardi S Malagola M Zanaglio C Polverelli N Dereli Eke E D’Adda M. Digital PCR improves the quantitation of DMR and the selection of CML candidates to TKIs discontinuation. Cancer Med (2019) 8:2041–55. doi: 10.1002/cam4.2087 PMC6536984 30950237

[50] Bernardi S Foroni C Zanaglio C Re F Polverelli N Turra A. Feasibility of tumor−derived exosome enrichment in the onco−hematology leukemic model of chronic myeloid leukemia. Int J Mol Med (2019) 44:2133–44. doi: 10.3892/ijmm.2019.4372 PMC6844640 31638195

[51] Ohmine K Ota J Ueda M Ueno S Yoshida K Yamashita Y. Characterization of stage progression in chronic myeloid leukemia by DNA microarray with purified hematopoietic stem cells. Oncogene (2001) 20:8249–57. doi: 10.1038/sj.onc.1205029 11781839

[52] Oehler VG Yeung KY Choi YE Bumgarner RE Raftery AE Radich JP. The derivation of diagnostic markers of chronic myeloid leukemia progression from microarray data. Blood (2009) 114:3292–8. doi: 10.1182/blood-2009-03-212969 PMC2759651 19654405

[53] Flamant S Ritchie W Guilhot J Holst J Bonnet M-L Chomel J-C. Micro-RNA response to imatinib mesylate in patients with chronic myeloid leukemia. Haematologica (2010) 95:1325–33. doi: 10.3324/haematol.2009.020636 PMC2913081 20460641

[54] Habib EM Nosiar NA Eid MA Taha AM Sherief DE Hassan AE. Circulating miR-146a expression predicts early treatment response to imatinib in adult chronic myeloid leukemia. J Investig Med Off Publ. Am Fed. Clin Res (2021) 69:333–7. doi: 10.1136/jim-2020-001563 33172871

[55] Habib EM Nosiar NA Eid MA Taha AM Sherief DE Hassan AE. MiR-150 expression in chronic myeloid leukemia: relation to imatinib response. Lab Med (2022) 53:58–64. doi: 10.1093/labmed/lmab040 34350970

[56] Li Y Wang H Tao K Xiao Q Huang Z Zhong L. miR-29b suppresses CML cell proliferation and induces apoptosis via regulation of BCR/ABL1 protein. Exp Cell Res (2013) 319:1094–101. doi: 10.1016/j.yexcr.2013.02.002 23428668

[57] Liu Y Song Y Ma W Zheng W Yin H. Decreased microRNA-30a levels are associated with enhanced ABL1 and BCR-ABL1 expression in chronic myeloid leukemia. Leuk. Res (2013) 37:349–56. doi: 10.1016/j.leukres.2012.12.003 23287430

[58] Chen N Meng Z Song J Kong L Zhang Y Guo S. miR-506 in patients with chronic myeloid leukemia and its effect on apoptosis of K562 cells. Am J Transl Res (2021) 13:9413–20. doi: 10.2217/fon-2018-0741 PMC8430173 34540060

[59] Zhang J Jiang Y Han X Roy M Liu W Zhao X. Differential expression profiles and functional analysis of plasma miRNAs associated with chronic myeloid leukemia phases. Future Oncol (2019) 15:763–76. doi: 10.2217/fon-2018-0741 30501399

[60] Mirza MAB Guru SA Abdullah SM Rizvi A Saxena A. microRNA-21 expression as prognostic and therapeutic response marker in chronic myeloid leukaemia patients. Asian Pac. J Cancer Prev APJCP (2019) 20:2379–83. doi: 10.31557/APJCP.2019.20.8.2379 PMC6852824 31450909

[61] Ohyashiki JH Ohtsuki K Mizoguchi I Yoshimoto T Katagiri S Umezu T. Downregulated microRNA-148b in circulating PBMCs in chronic myeloid leukemia patients with undetectable minimal residual disease: A possible biomarker to discontinue imatinib safely. Drug Des Devel. Ther (2014) 8:1151–9. doi: 10.2147/DDDT.S66812 PMC4149385 25187697

[62] Ohyashiki K Umezu T Katagiri S Kobayashi C Azuma K Tauchi T. Downregulation of plasma miR-215 in chronic myeloid leukemia patients with successful discontinuation of imatinib. Int J Mol Sci (2016) 17:570. doi: 10.3390/ijms17040570 27092489 PMC4849026

[63] Joshi D Chandrakala S Korgaonkar S Ghosh K Vundinti BR. Down-regulation of miR-199b associated with imatinib drug resistance in 9q34.1 deleted BCR/ABL positive CML patients. Gene (2014) 542:109–12. doi: 10.1016/j.gene.2014.03.049 24680705

[64] Zhang J Li S Li L Li M Guo C Yao J. Exosome and exosomal microRNA: trafficking, sorting, and function. Genomics Proteomics Bioinf (2015) 13:17–24. doi: 10.1016/j.gpb.2015.02.001 PMC4411500 25724326

[65] Russo D Garcia-Gutierrez JV Soverini S Baccarani M. Chronic myeloid leukemia prognosis and therapy: criticisms and perspectives. J Clin Med (2020) 9:1709. doi: 10.3390/jcm9061709 32498406 PMC7357035

[66] Mahon F-X Réa D Guilhot J Guilhot F Huguet F Nicolini F. Discontinuation of imatinib in patients with chronic myeloid leukaemia who have maintained complete molecular remission for at least 2 years: the prospective, multicentre stop imatinib (STIM) trial. Lancet Oncol (2010) 11:1029–35. doi: 10.1016/S1470-2045(10)70233-3 20965785

[67] Raza A Khan AQ Inchakalody VP Mestiri S Yoosuf ZSKM Bedhiafi T. Dynamic liquid biopsy components as predictive and prognostic biomarkers in colorectal cancer. J Exp Clin Cancer Res CR (2022) 41:99. doi: 10.1186/s13046-022-02318-0 35292091 PMC8922757

[68] Yu W Hurley J Roberts D Chakrabortty SK Enderle D Noerholm M. Exosome-based liquid biopsies in cancer: opportunities and challenges. Ann Oncol Off J Eur Soc Med Oncol (2021) 32:466–77. doi: 10.1016/j.annonc.2021.01.074 PMC8268076 33548389

[69] Bergantim R Peixoto da Silva S Polónia B Barbosa MAG Albergaria A Lima J. Detection of measurable residual disease biomarkers in extracellular vesicles from liquid biopsies of multiple myeloma patients-A proof of concept. Int J Mol Sci (2022) 23:13686. doi: 10.3390/ijms232213686 36430163 PMC9690807

[70] Asano M Umezu T Katagiri S Kobayashi C Tauchi T Gotoh M. Up-regulated exosomal miRNA-140-3p in CML patients with musculoskeletal pain associated with discontinuation of tyrosine kinase inhibitors. Int J Hematol (2017) 105:419–22. doi: 10.1007/s12185-017-2199-z 28197964

[71] Bouvy C Wannez A Laloy J Chatelain C Dogné J-M. Transfer of Multidrug Resistance among Acute Myeloid Leukemia Cells via Extracellular Vesicles and Their microRNA Cargo. Leuk. Res (2017) 62:70–6. doi: 10.1016/j.leukres.2017.09.014 28987820

[72] Min Q-H Wang X-Z Zhang J Chen Q-G Li S-Q Liu X-Q. Exosomes derived from imatinib-resistant chronic myeloid leukemia cells mediate a horizontal transfer of drug-resistant trait by delivering miR-365. Exp Cell Res (2018) 362:386–93. doi: 10.1016/j.yexcr.2017.12.001 29223442

[73] Hershkovitz-Rokah O Modai S Pasmanik-Chor M Toren A Shomron N Raanani P. MiR-30e induces apoptosis and sensitizes K562 cells to imatinib treatment via regulation of the BCR-ABL protein. Cancer Lett (2015) 356:597–605. doi: 10.1016/j.canlet.2014.10.006 25305453

[74] Hrdinova T Toman O Dresler J Klimentova J Salovska B Pajer P. Exosomes released by imatinib−resistant K562 cells contain specific membrane markers, IFITM3, CD146 and CD36 and increase the survival of imatinib−sensitive cells in the presence of imatinib. Int J Oncol (2021) 58:238–50. doi: 10.3892/ijo.2020.5163 33491750

[75] Dong Y Lin Y Gao X Zhao Y Wan Z Wang H. Targeted blocking of miR328 lysosomal degradation with alkalized exosomes sensitizes the chronic leukemia cells to imatinib. Appl Microbiol Biotechnol (2019) 103:9569–82. doi: 10.1007/s00253-019-10127-3 31701195

[76] Lin H Rothe K Chen M Wu A Babaian A Yen R. The miR-185/PAK6 axis predicts therapy response and regulates survival of drug-resistant leukemic stem cells in CML. Blood (2020) 136:596–609. doi: 10.1182/blood.2019003636 32270193 PMC7485576

[77] Liu Y Song B Wei Y Chen F Chi Y Fan H. Exosomes from mesenchymal stromal cells enhance imatinib-induced apoptosis in human leukemia cells via activation of caspase signaling pathway. Cytotherapy (2018) 20:181–8. doi: 10.1016/j.jcyt.2017.11.006 29269240

[78] Chen X Chen Y Zhang M Cheng H Mai H Yi M. HucMSC Exosomes Promoted Imatinib-Induced Apoptosis in K562-R Cells via a miR-145a-5p/USP6/GLS1 Axis. Cell Death Dis (2022) 13:92. doi: 10.1038/s41419-022-04531-3 35091542 PMC8799639

[79] Yen R Grasedieck S Wu A Lin H Su J Rothe K. Identification of key microRNAs as predictive biomarkers of nilotinib response in chronic myeloid leukemia: A sub-analysis of the ENESTxtnd clinical trial. Leukemia (2022) 36:2443–52. doi: 10.1038/s41375-022-01680-4 35999259

[80] Liu J Zhang Y Liu A Wang J Li L Chen X. Distinct dasatinib-induced mechanisms of apoptotic response and exosome release in imatinib-resistant human chronic myeloid leukemia cells. Int J Mol Sci (2016) 17:531. doi: 10.3390/ijms17040531 27070592 PMC4848987

[81] Bellavia D Raimondo S Calabrese G Forte S Cristaldi M Patinella A. Interleukin 3- receptor targeted exosomes inhibit *in vitro* and *in vivo* chronic myelogenous leukemia cell growth. Theranostics (2017) 7:1333–45. doi: 10.7150/thno.17092 PMC5399597 28435469

[82] Taverna S Fontana S Monteleone F Pucci M Saieva L De Caro V. Curcumin Modulates Chronic Myelogenous Leukemia Exosomes Composition and Affects Angiogenic Phenotype via Exosomal miR-21. Oncotarget (2016) 7:30420–39. doi: 10.18632/oncotarget.8483 PMC5058690 27050372

[83] Raimondo S Naselli F Fontana S Monteleone F Lo Dico A Saieva L. Citrus limon-derived nanovesicles inhibit cancer cell proliferation and suppress CML xenograft growth by inducing TRAIL-mediated cell death. Oncotarget (2015) 6:19514–27. doi: 10.18632/oncotarget.4004 PMC4637302 26098775

[84] Cochran AM Kornbluth J. Extracellular vesicles from the human natural killer cell line NK3.3 have broad and potent anti-tumor activity. Front Cell Dev Biol (2021) 9:698639. doi: 10.3389/fcell.2021.698639 34368150 PMC8343581

[85] Samara A Anbar M Shapira S Zemlyansky A Zozovsky A Raanani P. Using natural killer cell-derived exosomes as a cell-free therapy for leukemia. Hematol Oncol (2022) 41(3):487–498. doi: 10.1002/hon.3111 36451254

[86] Zhang X Yang Y Yang Y Chen H Tu H Li J. Exosomes from bone marrow microenvironment-derived mesenchymal stem cells affect CML cells growth and promote drug resistance to tyrosine kinase inhibitors. Stem Cells Int (2020) 2020:8890201. doi: 10.1155/2020/8890201 33414831 PMC7752271

[87] Houshmand M Garello F Stefania R Gaidano V Cignetti A Spinelli M. Targeting chronic myeloid leukemia stem/progenitor cells using venetoclax-loaded immunoliposome. Cancers (2021) 13:1311. doi: 10.3390/cancers13061311 33804056 PMC8000981

